# Is Previous Disaster Experience a Good Predictor for Disaster Preparedness in Extreme Poverty Households in Remote Muslim Minority Based Community in China?

**DOI:** 10.1007/s10903-012-9761-9

**Published:** 2012-12-22

**Authors:** Emily Y. Y. Chan, Jean H. Kim, Cherry Lin, Eliza Y. L. Cheung, Polly P. Y. Lee

**Affiliations:** Collaborative Centre for Oxford University and CUHK for Disaster and Medical Humanitarian Response, Room 308, The Jockey Club School of Public Health and Primary Care, Faculty of Medicine, The Chinese University of Hong Kong, Hong Kong, China

**Keywords:** Disaster preparedness, Extreme poverty household, Ethnic minority, Rural and remote communities, China

## Abstract

Disaster preparedness is an important preventive strategy for protecting health and mitigating adverse health effects of unforeseen disasters. A multi-site based ethnic minority project (2009–2015) is set up to examine health and disaster preparedness related issues in remote, rural, disaster prone communities in China. The primary objective of this reported study is to examine if previous disaster experience significantly increases household disaster preparedness levels in remote villages in China. A cross-sectional, household survey was conducted in January 2011 in Gansu Province, in a predominately Hui minority-based village. Factors related to disaster preparedness were explored using quantitative methods. Two focus groups were also conducted to provide additional contextual explanations to the quantitative findings of this study. The village household response rate was 62.4 % (n = 133). Although previous disaster exposure was significantly associated with perception of living in a high disaster risk area (OR = 6.16), only 10.7 % households possessed a disaster emergency kit. Of note, for households with members who had non-communicable diseases, 9.6 % had prepared extra medications to sustain clinical management of their chronic conditions. This is the first study that examined disaster preparedness in an ethnic minority population in remote communities in rural China. Our results indicate the need of disaster mitigation education to promote preparedness in remote, resource-poor communities.

## Background

Disaster preparedness is an important disaster mitigation strategy to protect human lives for adverse human health impact of unforeseen disasters. Although studies indicated that degree of disaster preparedness in urban population of high income countries was positively associated with previous disaster exposure [7], little is known regarding remote populations, particularly those living in rural communities [4]. Previous studies indicated that rural residents were found to be more vulnerable, had a higher likelihood of receiving inadequate housing and were less able to manage a chronic ailment after an earthquake [8, 9]. Although these findings indicated that greater attention to pre-disaster preparedness and health services may be needed in rural communities, there are few published studies that investigate the predictors of disaster preparedness in remote, resource poor communities.

China is the most natural disaster-prone country globally [5]. Its remote geographic areas are disaster-prone and are predominantly comprised of ethnic minorities who are living under the World Bank definition of extreme poverty (<USD$1.25/person/day) [10]. A multi-site based ethnic minority project (2009–2015) is set up by an international disaster research collaboration which seeks to identify cost-effective strategies for improving resilience to natural disasters and health risk in remote, rural and disaster prone communities in China [2]. The Chinese Hui population, a Muslim ethnic minority group, resides in the Northwest area of China in the high plateau of the Yellow river. Since 2009, this Hui region has been studied by the research team.

This geographic area of the study site is known for its high risk to multitude large number of natural disasters, ranging from rainstorms (2005), extreme snowstorms (2007), earthquakes (2008), community fires (2009), drought (2010) and flooding (2010). The main objective for this study is to examine if previous disaster experience significantly increases household preparedness levels in an ethnic minority community residing in a remote, resource-poor area of China.

## Methods

A cross-sectional, cluster sampling, face-to-face household-based survey was conducted in Datan Village, Gansu province between 3rd and 9th Jan 2011. Two focus group were also conducted to provide additional contextual explanations to the quantitative findings of this study.

### Study Site

Datan Village is non-Han Chinese, Hui minority-based village near Tianshui City, Gansu province located at the high plateau of Yellow River. This village is comprised of four sub-villages, with 213 households and a total population size of 1 108.

### Data Collection Tools

The data collection tools include disaster preparedness questionnaire and focus group questions. The content validity of the survey questions was developed based on literature review and qualitative interviews at previous study result of the focus group studies in Ethnic Minority Health Project [2]. The survey intends to explore key information required to understand level of disaster preparedness. The study questionnaire had been language translation (Chinese–English–Chinese) and the final questionnaire was piloted and tested by the principal investigators on potential participants and revised accordingly.

### Disaster Preparedness Questionnaire

A disaster preparedness questionnaire collected information from four main areas. These included: (1) socio-demographic status (e.g. age, gender, occupation, average annual income, education level. Information about household size and number of children below 5 years old and older people above 60; chronic disease status and related medication use); disaster risk perceptions (Do you consider yourself living in a disaster high risk area?); (2) previous direct exposure to disaster (Have you ever experienced disaster in your lifetime?); (3) physical and psychological impacts of and concerns about disasters were also asked (Were you physically harmed during the previous disaster? Were you worried during the disaster? Do you think you have the ability to protect yourselves and your family’s safety in the future if disaster comes again?); (4) Disaster health and public health preparedness. Perception of preparedness was categorized into a Likert scale with 5 point ratings (1 = very well prepared, 2 = more prepared than other villagers, 3 = similar to others, 4 = less prepared than other villagers, 5 = no preparation at all); and (5) actual disaster preparation (Do you consider your household to be prepared for disaster? Do you have a readily available disaster emergency kit? Have you ever received tetanus vaccination before? Do you have emergency medicines available at home? Do you have enough money or resources to buy the medications that you need?). The study tool was tested and validated with local population.

### Focus Group Questions

Semi-structured guided questions were used for focus group investigation. The general context of the study site, such as physical, mental and social health, access to materials and health services were asked. In addition, questions related to disaster preparedness included “Do you consider yourself is living/not living in a disaster high risk area? If yes, why?”, “Have you ever experienced disaster in your lifetime?”, “Have you considered preparing a disaster emergency kit? If not, why?”, “What is/are the factor(s) for you to prepare/not prepare a disaster emergency kit?”, and “Have you prepared any medicines? What is/are the reasons for not preparing it?”.

### Data Collection

Data was collected using cluster sampling method. Through facilitation of Datan village chief, a representative of all the households from each of the four sub-villages was invited to participate in a health and disaster preparedness presentation in front of the village’s mosque (Day one for Village 1 and 2, Day two for Village 3 and 4). Upon arrival to the health talk, participants were invited to participate in the study. All participants were ensured about their right to attend the health talk even if they decided not to join the study, had the right to leave the study anytime and all questionnaires were anonymous. For those who agreed to join the study, survey forms were distributed. Study participants would then be interviewed individually and survey was filled out by one of the five trained research team members through the language support of three local translators. Each survey took about 20–30 min to complete. A disaster preparedness pack (with an emergency whistle and torch in a pouch) was given to every participant of study and the health talk upon completion.

To obtain a better qualitative understanding of disaster experiences, preparedness and challenges, two focus groups were also conducted with the study population. One focus group was designated for male participants and one for female participants. Participants were recruited by the head of village and their participation was entirely voluntary. In sum, 10 males (with the mean age of 45.2) and 11 females (with the mean age of 42) participated in the focus group. The major ethnicity is Hui for both male and female participants. Most female participants are illiterates and male participants had education ranged from illiterate to tertiary education. Most of them worked as farmers. The focus groups were conducted in the head of village’s household on the 11 Jan 2011. All the groups were audio-recorded for reporting purposes and all participants received a small gift of a toothbrush and toothpaste at the conclusion of the focus group.

The respondents of the survey group and focus group were different although the same household may have had a participant who joined the focus group study and another household member who joined the survey.

### Data Management and Statistical Analysis

All data were double-entered and descriptive statistics were calculated for the questionnaire items. Perception of disaster risk, prior disaster experience, self-efficacy in protecting oneself and safety of family members during disaster, ownership of a readily available disaster emergency kit, medicines, self-reported tetanus immunization status were re-coded as dummy variables (1 = yes, 0 = no). Chi square tests were performed between status of previous disaster exposure and (1) disaster risk perception, (2) self-efficacy in protecting safety during disaster, and (3) actual disaster preparedness behaviors (disaster emergency kit, preparation of medicines, and tetanus vaccination). For variables with *P* value <0.05 in the unadjusted analyses, logistic regression was then performed for variables. All statistical analysis was conducted with SPSS, version 16.0 (SPSS Inc., Chicago, USA).

The focus group data was transcribed and examined to enrich additional information and to provide clarification on the survey results. The data was examined to understand the underlying reasons for the different levels of disaster preparedness. The focus group data was transcribed and examined to help refine the study questionnaire, and to provide clarification on the survey results. The data was examined to understand the underlying reasons for the different levels of disaster preparedness. Content themes selected for overview included: “Why are you considering yourself living/not living in a disaster high risk area?”, “Have you considered preparing a disaster emergency kit? If not, why?”, “What is/are the factor(s) for you to prepare/not prepare a disaster emergency kit?”, and “Have you prepared any medicines? If not What is/are the reasons for not preparing it?”.

## Ethics Approval

Ethics approval was obtained from the Survey and Behavioral Research Ethics Committee of Chinese University of Hong Kong. Verbal consent was obtained from all participants.

## Results

The village study household participation response rate was 62.4 % (133/213) and 133 completed questionnaires were obtained. Table 1 shows a general description of demographic information of respondents when compared with general population of Gansu province [1] and general population of China [3]. All respondents were classified as living in extreme poverty (<USD$1.25/per day/per person) and were significantly lower ($247.24RMB) as compared with provincial and national data [6]. About 60 % of respondents were illiterate (>3 times the provincial illiteracy rate and >8 times the national average). A large proportion of the sample was farmers or fishermen. The average number of people in the household was slightly more than provincial and national data. For self-reported non-communicable diseases status, arthritis, hypertension and gastroenteritis were ranked as the most common non-communicable diseases among the study sample. In this study, the ratio of male to female respondents was slightly higher than that of Gansu provincial and national sample.

**Table 1:** Comparative demographic information of our study sampled population 2008 general provincial population^a^ and 2008 national sample of China population^b^

	Hui minority study sample (n = 133)	General population in the province^a^	China population^b^
Male life expectancy in year 2000	N/A	66.8 years	69.6 years
Female life expectancy (year 2000)	N/A	68.3 years	73.3 years
Average Age	43.0 years	NA	
Male to female gender ratio	1.22:1	0.98:1	1.06:1
Mean household income/year	1422.64 RMB	NA	NA
Mean per capita income/year	247.24 RMB	2772 RMB (rural only)	6701 RMB
% living <USD$1.25/day	100	12.7 (rural only)	15.9
Education level (by rank) [%]			
Illiterate	59.2	20.1	7.5
No formal ed. (but literate)	4.8	NA	
Primary school	25.6	NA	31.17
Jr. secondary school	7.2	NA	40.94
Sr. secondary/technical ed.	3.2	NA	13.69
College and above	0	NA	6.7
Occupation (%)			
Farmer and fisherman	86.6	NA	3.15
Non-agricultural worker	1.6	NA	41.91
Housewife	3.9	NA	NA
Unemployed	1.6	23	
Retired	3.9	NA	4.2
Others	2.4	NA	NA
Household composition			
Mean household size	5.7	3.3 people	4.01
Number of children <5 (%)			
1 child	53.3	NA	NA
2 children	30	NA	NA
≥3 children	16.7	NA	NA
Households with >1 elderly	52.9 % (>60 years)	11.6 % (>65 years)	NA
Non-communicable diseases (NCD)			
1st Most common reported NCD	Arthritis (26.4 %)	Hypertension (36.3 %)	NA
2nd Most common reported NCD	Hypertension (22.6 %)	Gastroenteritis (18.9 %)	NA
3rd Most common reported NCD	Gastroenteritis (18.9 %)	Gall stones/cholangitis (9.3 %)	NA

^a^2008 Gansu provincial data (based on sample size of 12 974); ^b^ general China information (obtained from the 2008 national sample survey). *NA* no available data. 1 RMB ≈ 0.15 USD

The self-reported disaster risk perceptions and preparedness of the respondents are shown in Table 2. Even though all respondents were living in areas with similar disaster risk, results indicated that previous direct disaster experiences were significantly associated with perception of disaster risk (OR = 6.16: 95 % CI 1.96–19.39). No significant association, however was found between self-reported previous disaster experience and respondent’s self-perceived disaster preparedness level, the respondent’s self-efficacy in protecting themselves, the respondent’s self-efficacy in protecting their family in the event of a major disaster.

**Table 2:** Self-reported disaster perception and preparedness (n = 126)

	% in total sample	No disaster experience (n = 26) % Referent group	Direct disaster experience (n = 100) % OR (95 % CI)	*p* value
Disaster-related perceptions				
Self-reporting perception of near-term disaster risk	47.5 % (57/120)	16.7 % OR = 1.00	55.2 % (53/96) 6.16 (1.96 –19.39)	**<0.01**
Self-reported perception of preparedness ^a^ Mean (SD)	2.82 (1.02)	2.94 (1.0)	3.28 (1.2)	0.31
Believing oneself to have self efficacy in protecting own safety	59.1 % (69/117)	59.1 % OR = 1.00	58.9 %^b^(56/95) 0.99 (0.39–2.55)	0.99
Believing oneself to have self Efficacy in protecting family’s safety	47.7 % (53/111)	47.6 % OR = 1.00	47.8 % (43/90) 1.01 (0.39–2.61)	0.99
Disaster preparedness				
Possess a disaster emergency kit in household	10.7 % (13/121)	4.2 % OR = 1.00	12.4 % (12/97) 3.25 (0.40–26.29)	0.46
Have drugs prepared in event of emergency or disaster	28.9 % (33/114)	21.7 % OR = 1.00	30.8 % (28/91) 1.60 (0.54–4.74)	0.45
Have drugs prepared for non-communicable disease conditions (among households that included member(s) with NCD)	9.6 % (5/52)	8.3 % OR = 1.00	10.0 % (4/40) 1.22 (0.12–12.11)	0.99
Have had tetanus vaccination^b^	20.4 % (22/108)	10.0 % OR = 1.00	22.7 % (20/88) 2.65 (0.57–12.394)	0.36

^a^Perception of preparedness was categorized into a Likert scale with 5 point ratings (1 = very well prepared, 2 = more prepared than other villagers, 3 = similar to others, 4 = less prepared than other villagers, 5 = no preparation at all). *t* test was performed for this analysis. ^b^ No public data was available for tetanus vaccination statistic of National population except the rate of diphtheria, pertussis and tetanus (DPT) vaccination of 1-year-old children was 99 % 2007.^9^

In terms of personal health preparedness of the study community, tetanus immunization coverage in the community was 20.4 % and was unrelated to previous exposure to disaster. At the household level, only 10.7 % reported to have a readily available disaster emergency kit at home.

If either owning a readily available—disaster emergency kit, stocking up with emergency and non-communicable drugs or obtaining tetanus vaccination might be used as a proxy to represent disaster preparedness, study findings indicated that no increase association was found among any of these reported preparedness efforts with direct disaster experience. In addition, although 90.8 % of the participants considered it important to bring medications along in the event of a disaster, only 30.3 % had medications readily available at home at the time of the survey.

Table 3 presented the reasons reported by the respondents for not having a disaster kit or regularly used medications readily available at home. The main reason for not having a disaster kit at home was “never occurred to have one” (42.7 %).

**Table 3:** Self-reported reasons for lack of disaster preparation

Self-report reasons given by respondents	%
For not preparing a disaster kit (n = 82)	
“Never occurred to have one”	42.7
“Wanted to but lacked the resource to do so”	31.7
“Did not consider it important or necessary”	10.7
“Didn’t have the time to implement”	9.8
Others	5.1
For not preparing medications (n = 54)	
“Wanted to but lacked the resource to do so”	43.6
“Never occurred to have one”	32.7
“Didn’t have the time to implement”	9.1
“Did not consider it important or necessary”	5.5
Others	9.1

Of the sample, 43.6 % reported “lack of resources” as the primary reason for not having regularly used medications in the household. However, nearly one-third stated that they did not have such drugs on hand because they had not previously thought about preparing such resources to mitigate the adverse impact of disaster. 14.6 % reported not having prepared medications because they did not have time nor did not perceive the necessity. These findings indicated the potential value and need for disaster preparedness education in these communities. The qualitative data uncovered additional explanations for lack of disaster preparedness. Some respondents perceived these types of preventive measures to be ineffective in mitigating the effects of large-scale natural disasters.

In summary, findings indicated previous direct disaster experience was associated with risk perception. However, contrary to previous studies in western populations, disaster experience was found not to be associated with disaster preparedness. The lack of awareness and lack of pre-contemplation appear to be important factors that may contribute to the low level of disaster preparedness in rural, resource-poor communities.

## Discussion

The results revealed a limited level of disaster preparedness in the extreme poverty ethnic minority village in rural China. Although the population had experienced multiple disasters, few villagers in Datan village possessed specific disaster-related plans or preparedness. Of the villagers surveyed, 25 % of the study respondents reported practically no disaster preparedness at all. Moreover, as highlighted in the findings, more than 50 % of respondents had not contemplated preparing a disaster kit or drugs for future disasters. Findings indicated previous disaster exposure was shown to be an indicator for individuals’ self-perceived risk of living in a disaster prone area but failed to differentiate self-efficacy level for disaster related actions.

Limitations of this study include possible reporting bias and recall bias. Our study had local community support and had used three translators to interview the large proportion of respondents who did not speak Mandarin Chinese. There still exists the possibility of differential interpretation of the questions and responses in the respondents speaking the local dialect.

In addition, as this is a cross-sectional study of self-reported behaviors and perceptions, it is difficult to ascertain the causal direction between disaster preparedness and disaster experience. Although this study focuses only on the Hui minority group and the generalizability of findings to other minority-based communities with different beliefs and practices may be limited, this is the first study reporting public health disaster preparedness in an ethnic minority area in China. Findings may serve to provide better understanding of rural community risk perception. These insights may be used to develop health education and interventions for mitigating adverse human impact of disasters.

Our findings suggest that preparedness education may be a cost-effective approach for raising the awareness and preparedness level in residents of remote, rural areas. Emphasizing the importance of household-based preparedness and providing basic resources to these impoverished communities may better protect these remote communities from adverse health impacts of disaster.

## Conclusion

Although risk perceptions may be associated with previous disaster exposure, the study findings indicate that in remote and resource-poor areas, disaster preparedness may not be associated with previous disaster experiences. Further studies will be necessary to understand the motivation and to ascertain the optimal strategies for improving disaster preparedness in remote communities.

## Key Messages

- Although literature indicated prior direct experience in natural disaster is associated with heighten disaster risk perception and preparedness in western urban communities, this study indicated that direct previous disaster experience was not associated with better disaster preparedness in extreme poverty households in remote China.
- Despite previous disaster exposure was significantly associated with perception of living in a high disaster risk area (OR = 6.16: 95 % CI 1.96–19.39), only 10.7 % households possessed a disaster emergency kit. Among the household with members who had non-communicable diseases, about 9.6 % prepared extra medications to sustain clinical management of their chronic conditions.
- Among respondents, 59 % reported confidence in protecting themselves against the adverse impact of disaster, but less than 50 % felt self-efficacy in protecting the household’s safety during disaster.
- About 11 % of the households reported having an emergency kit and 10 % reported that they had chronic disease medication ready in response to emergency needs. The two main reasons for minimal disaster preparation were lack of resources and awareness.
- Disaster preparedness education should be promoted and supported in remote, rural, extreme poverty Chinese communities.
